# The Role of Natural Killer Cells and Their Metabolism in HIV-1 Infection

**DOI:** 10.3390/v16101584

**Published:** 2024-10-09

**Authors:** Kewreshini K. Naidoo, Marcus Altfeld

**Affiliations:** 1 Department of Virus Immunology, Leibniz Institute of Virology, 20251 Hamburg, Germany; 2 German Center for Infection Disease (DZIF), Partner Site Hamburg-Lübeck-Borstel-Riems, 20251 Hamburg, Germany; 3 Institute of Immunology, University Medical Center Hamburg-Eppendorf, 20251 Hamburg, Germany

**Keywords:** HIV-1 infection, innate immunity, antiviral immunity, natural killer cells, immunometabolism, NK cell metabolism

## Abstract

Natural killer (NK) cells are multifaceted innate effector cells that critically influence antiviral immunity, and several protective NK cell features that modulate HIV-1 acquisition and viral control have been described. Chronic HIV-1 infection leads to NK cell impairment that has been associated with metabolic dysregulations. Therapeutic approaches targeting cellular immune metabolism represent potential novel interventions to reverse defective NK cell function in people living with HIV.

## 1. Introduction

Natural killer (NK) cells are immune cells of lymphoid lineage that were initially described for their distinctive “spontaneous” cytotoxicity with no apparent need for priming or prior sensitization [1], and as such were assigned to the innate arm of immunity. Years of intensive research have debunked their reputation as mere killers and exposed functional complexity far beyond initial assumption. The antitumor and antiviral activities of NK cells are mediated through cytotoxic programmes, principally the release of cytolytic granules and the expression of death ligands, which lead to the induction of cell death pathways in target cells. The importance of these cytotoxic mechanisms is particularly apparent in persons with NK cell deficiencies or functional defects that present with recurrent infection [2,3]. Moreover, NK cells contribute to inflammation and exhibit immunoregulatory attributes that shape innate and adaptive immunity.

NK cells constitute 5–15% of peripheral blood mononuclear cells under homeostatic conditions. Common identification is based on the lack of CD3/CD19 and expression of the adhesion molecule CD56, with around 90% of NK cells expressing CD16 (Fc*γ*RIIIa). The relative surface expression of CD56 molecules further distinguish NK cells as CD56^dim^ or CD56^bright^ [4,5]. In blood, CD56^dim^ and CD56^bright^ NK cells account for approximately 90% and 10% of absolute NK cells, respectively. Subset proportions are reversed within secondary lymphoid tissues where CD56^bright^ NK cells preferentially reside [6]. CD56^dim^ and CD56^bright^ NK cell subsets are functionally distinct; the CD56^dim^ NK cell subset exhibits robust cytotoxicity, whereas CD56^bright^ NK cells are potent cytokine producers with higher proliferation potential. A third NK cell subset, CD56^neg^ cells, are rare in healthy individuals, but expand during chronic viral infection at the expense of CD56-expressing NK cells. The CD56^neg^ NK cell subset has been shown to be functionally impaired, either lacking or exhibiting defective effector capabilities [7]. Although NK cell subset classification is commonly based on the relative expression of CD56 molecules, other established designations pertain to location (circulating vs. tissue-resident), the expression of inhibitory NK cell receptors (uneducated vs. educated) and, most recently, on energy metabolism (basal vs. activated) [8,9].

NK cells do not express antigen-specific receptors; instead, differentially expressed, germline-encoded surface molecules facilitate the detection of virus-infected or transformed target cells. These surface receptors are either NK cell exclusive or expressed broadly on hematopoietic lineage cells; and principally comprise of killer-cell immunoglobulin-like receptors (KIRs), C-type lectins (NKG2), natural cytotoxicity receptors (NCRs) and the CD16 receptor [10]. KIRs are transmembrane glycoproteins that are either activating or inhibitory in nature and are distinguished by the length and structure of the cytoplasmic tail. Activating KIRs have short cytoplasmic tails that, via transmembrane lysine residues, interact with immunoreceptor tyrosine-based activation motif (ITAM)-containing adaptor proteins; whilst inhibitory KIRs have long cytoplasmic tails with two immunoreceptor tyrosine-based inhibition motifs (ITIMs) that upon ligand interaction recruit phosphatases SHP-1 and SHP-2 [10,11]. Activating NK cell receptors (NKRs), consisting of activating KIRs, NCRs (NKp30, NKp44 and NKp46), CD94/NKG2C heterodimer, NKG2D and the coactivating/adhesion DNAX-activating molecule (DNAM-1), recognise and directly bind to pathogen-derived or stress-induced antigens expressed on target cells [12]. Inhibitory NKRs, including inhibitory KIRs, lymphocyte-activation gene 3 (LAG-3), T cell immunoreceptor with Ig and ITIM domains (TIGIT), T cell immunoglobulin and mucin domain-containing protein 3 (Tim-3) and the CD94/NKG2A heterodimer, convey signals of health through engagement with self-ligands, specifically human leukocyte antigen (HLA) molecules, which counteract activation signalling. During immune surveillance, NK cells engage in numerous receptor-ligand interactions, each with a unique signalling transduction. The overall responsiveness of NK cells is governed by a balanced integration of activating and inhibitory signals [13].

The maturation and functional capacity of an individual NK cell is established during the development process of NK cell education or licensing. Specific NKRs, predominantly inhibitory KIR, interact with cognate HLA ligands expressed on adjacent self-cells. KIR and HLA genes segregate independently and together constitute the most polymorphic and polygenic coding regions within the human genome. Thus, allelic variation contributes to extensive repertoire diversity of both KIR and HLA at the phenotypic level, influencing the avidity of receptor-ligand pair interactions that ultimately define the reactivity threshold and potency of individual NK cell clones. NK cells lacking surface inhibitory NKR expression for licensing are largely hyporesponsive and require robust activation signalling to trigger reactivity. The NK cell education process confers functional competence and a capacity to distinguish between self and non-self [14,15,16]. Although NK cells are confined to the expression of germline-encoded receptors, the unique combinations of receptor expression on individual NK cell clones contribute to a diverse NK cell repertoire with broad response potential; and further complexity effected by local environmental factors, including the inflammatory milieu, metabolic mediators and non-self-stimuli, engender an NK cell population with extensive functional heterogeneity [17,18].

## 2. NK Cell-Mediated Immunity to HIV-1 Infection and Viral Evasion Mechanisms

Multiple epidemiological and mechanistic studies attest to the protective features of NK cell immunity in HIV-1 infection that is evident in both resistance to HIV-1 acquisition and viral containment following transmission. Since NK cell reactivity is tightly controlled through the engagement of surface KIR with their cognate HLA molecules, it is no surprise that certain KIR/HLA combined genotypes have been associated with HIV-1 infection outcomes [19,20]. Additionally, NK cell defence mechanisms include chemokine production and antibody-dependent cellular cytotoxicity (ADCC). Several reports describe that HIV-1 has evolved mechanisms to evade NK cell recognition [21], which only emphasises the importance of these NK cell-antiviral responses in the immune control of HIV-1 infection.

### 2.1. KIR/HLA-Mediated Mechanisms

#### 2.1.1. The ‘Missing Self’ Mechanism

**Figure 1:**
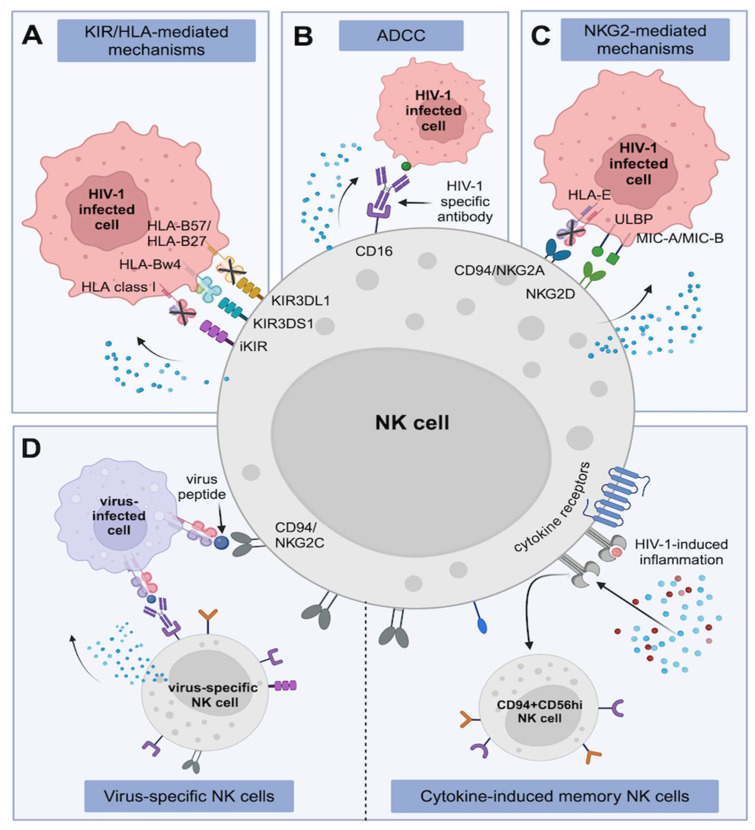
NK cell-mediated immunity in response to HIV-1 infection. (**A**) HIV-1-induced downregulation of HLA class I molecules render HIV-1-infected cells vulnerable for killing by licensed NK cells. Certain KIR/HLA combinations are also associated with protection against HIV-1 acquisition or NK cell-mediated control during HIV-1 infection. (**B**) NK cell-mediated antibody-dependent cellular cytotoxicity (ADCC) activity promotes potent lysis of HIV-1-infected cells and is associated with better clinical outcomes in HIV-1 infection. (**C**) Downmodulation of HLA-E on HIV-1-infected cells makes them susceptible to killing by NKG2A-expressing NK cells. NKG2D receptors recognise and bind virus-derived cell surface molecules on HIV-1-infected cells for targeted NK-cell mediated killing. (**D**) NK cell CD94/NKG2C interaction with antigen-expressing HLA-E on virus-infected cells drives the expansion of virus-specific memory NK cells with superior ADCC activity in HCMV and HIV-1 infection. Memory-like NK cells can also be cytokine-induced during inflammation.

HIV-1 proteins can target and downregulate the surface expression of HLA class I molecules in an attempt to evade cytotoxic T cell recognition and killing, but the absence of HLA-expression render HIV-1-infected target cells vulnerable for killing by licensed NK cells. Viral mechanisms to evade this NK cell-mediated killing involve only partial or incomplete HLA downmodulation, while conserving or upregulating expression of ligands that engage inhibitory NKRs. The HIV-1 Nef protein binds HLA-A and HLA-B at the cytoplasmic region, impeding molecule expression at the cell membrane level [22,23,24]. Selective HLA-A and HLA-B downmodulation restricts virus-specific CD8+ T cell activity, while maintenance of HLA-C and HLA-E expression provides ligands for several inhibitory KIRs, particularly of the KIR2DL family, protecting virus-infected cells from KIR2DL-educated NK cells [22,25]. In comparison, different KIR3DL1 isoforms interact with HLA-B molecules carrying the Bw4 motif, with consequences for receptor-ligand binding avidity and NK cell education [26,27], thus KIR3DL1/HLA-Bw4 educated NK cells, in the absence of HLA-B expression and the presence of stress-induced receptors, are recalibrated toward an activated antiviral state [28]. Reduced HLA class I surface expression is also induced through HIV-1 Tat protein-mediated repression of the β_2_-microglobulin promoter [29], whilst the Vpu protein facilitates the downregulation of HLA-C molecules [30,31,32]. HLA-C1 and HLA-C2 isoforms are recognised by inhibitory KIR2DL receptors [33,34,35]. Although the strength of binding avidity and consequences for NK cell education of different KIR2DL/HLA-C isoforms remain incompletely understood and strongly depend on the respective HLA-C and KIR2DL genotypes, loss of surface HLA-C expression does prompt NK cell-mediated activity of KIR2DL-educated NK cells against autologous HIV-1-infected CD4+ T cell targets [32] (Figure 1A). Taken together, these data highlight the impact of genetic variation in inhibitory KIR receptors and their HLA class I ligands for determining NK cell activation thresholds via licensing and the consequences for the potency of antiviral NK cell responses.

#### 2.1.2. Protective and Deleterious KIR/HLA Mechanisms

The first descriptions implicating KIR3DS1/KIR3DL1 and HLA-Bw4 compound genotypes on HIV-1 disease outcomes emanated from epidemiological analyses of people living with HIV (PLWH) enrolled in the Multicenter AIDS Cohort Study [36,37]. Combined KIR/HLA genotypes, specifically KIR3DL1 in combination with HLA-Bw4, co-carriage of KIR3DL1-high and KIR3DL1-low alleles with HLA-B*57 and HLA-B*27, and the synergistic effect of activating KIR3DS1 together with HLA-Bw4-80I (encoding for isoleucine at position 80) were associated with slower time to the onset of acquired immunodeficiency syndrome (AIDS) [36], and lowered the risk of certain opportunistic infections [38]. The protective feature of the KIR3DS1/HLA-Bw4-801 pairing was attributed to the preferential activation and expansion of KIR3DS1+ NK cells that strongly suppressed HIV-1 replication in HLA-Bw4-80I infected target cells in a dose and cell contact dependent manner [39,40]. NK cells with at least one copy of the KIR3DS1 gene exhibited enhanced degranulation and IFN-γ responses in acute phase HIV-1 infection, in particular in individuals also encoding for HLA-B*57 and B*58 [41] (Figure 1A). Findings from the Multicenter AIDS Cohort Study raised the question of possible protective KIR/HLA genetic pairings that account for the superior NK cell activity observed in high-risk HIV-1-exposed seronegative (HESN) individuals.

HESN individuals remain HIV-uninfected despite frequent exposure to the virus. The identification of people with presumed resistance to HIV-1 infection sparked considerable interest within several research groups [42] and it was apparent, even from earlier studies, that the correlates of protection were not solely attributable to adaptive immunity [43,44,45,46,47,48,49,50]. Hints of NK cell-mediated protection initially emerged from a cohort of HIV-1-exposed Vietnamese intravascular drug users (IDUs) [51]. In functional analyses, NK cells in HESN IDUs exhibited superior cytolytic and cytokine responses in comparison to NK cells in IDUs living with HIV, prior to and following seroconversion [51]. Similarly, enhanced NK cell-mediated IFN-γ responses correlated with lower risk of HIV-1 acquisition in high-risk females [52]. NK cell-mediated resistance to HIV-1 in HESN individuals have thus far been associated with specific KIR with or without combinational HLA genotypes that are more frequent in HESN than in HIV-1-susceptible persons. In highly HIV-1-exposed African cohorts, inhibitory KIR genes, specifically KIR2DL2/KIR2DL3 heterozygosity and KIR3DL1 homozygosity, were more frequently present in HESN individuals without their cognate HLA class I ligands, HLA-C1 and HLA-Bw4, respectively [53]. In contrast, HIV-1-seropositive individuals encoded more frequently for inhibitory KIR/HLA pairings (i.e., KIR2DL3 homozygosity/HLA-C1 and a tendency for KIR3DL1/HLA-Bw4 homozygosity) [53]. Additionally, co-carriage of the KIR3DL1 homozygous genotype (KIR3DL1*h/*y) together with HLA-B*57 was more frequent in HESN individuals compared to persons with acute HIV-1 infection (AHI) [54]. NK cells derived from carriers of the KIR3DL1*h/*y/HLA-B*57 gene combination displayed higher polyfunctional potential in response to HLA-devoid target cells than carriers of other KIR3DL1*h/*y/HLA-B pairings [55,56]. Importantly, both HESN and AHI individuals carried similar frequencies of the KIR3DL1*h/*y genotype, but it was the combined presence of HLA-B*57 that was associated with HIV-1 resistance. Furthermore, homozygosity for activating KIR3DS1 was associated with lower HIV-1 infection risk [57] and delayed the time to HIV-1 seroconversion [58].

Paradoxically, certain KIR and KIR/HLA genotypes are also implicated in detrimental outcomes of HIV-1 disease. KIR3DS1, in the absence of HLA-Bw4-80I, has been associated with more rapid disease progression [36]. Similarly, other activating KIR, namely KIR2DS2 and KIR2DS4, were linked to either faster CD4+ T cell decline or higher relative viremia, predictive of accelerated progression to AIDS [59,60,61]. Furthermore, higher mortality rates were associated with KIR2DL3/HLA-C1 receptor–ligand co-carriage in PLWH [62]. These data suggest that NK cell populations skewed toward a more activated profile could evoke an exacerbated pro-inflammatory environment, contributing to chronic immune activation and accelerated HIV-1 disease progression.

### 2.2. CD16-, NKG2A- and NKG2D-Mediated Regulation of Antiviral NK Cells

Engagement of the NK cell low-affinity CD16 receptor with opsonised membrane-bound target antigens promotes the potent lysis of virus-infected cells via antibody-dependent cellular cytotoxicity (ADCC) [4,63]. NK cell-mediated ADCC correlates with better clinical outcomes in HIV-1 infection, including a lower viral set point and higher CD4+ T cell counts [64,65], and has been associated with spontaneous viral control and slower progression of HIV-1 infection [66,67]. The description that NK cell-mediated ADCC activity was associated with modest protective efficacy in the RV144 HIV-1 vaccine trial [68] garnered widespread interest and accelerated research related to NK cell-mediated ADCC for HIV-1-immunotherapy approaches. Efficient HIV-1-specific ADCC has been linked to NK cell education and differentiation that facilitates enhanced antibody-dependent responsiveness [69,70]. Viral evasion mechanisms from ADDC activity have been described; for example, the HIV-1 accessory protein Vpu protects against ADDC-mediated clearance of virus-infected cells by downregulation and degradation of the anti-viral factor tetherin, with consequences for antibody recognition of virus-infected cells and NK cell-mediated ADCC [71,72,73] (Figure 1B).

The activating CD94/NKG2C and inhibitory CD94/NKG2A heterodimers interact with the non-classical HLA-E molecule, although dominant signalling is mediated via NKG2A engagement [74,75]. HLA-E expression is dependent on the binding of a signal peptide derived from the leader sequence of HLA class I molecules to HLA-E [76,77,78]. Primary HIV-1 strains can differentially modulate the surface expression of HLA-E molecules on infected CD4+ T cells through the Nef protein [79]. Lower HLA class I surface expression has been furthermore associated with downmodulation of HLA-E expression on infected cells, making them susceptible to killing by NKG2A-expressing NK cells that are normally inhibited through HLA-E [79,80,81]. The mechanisms by which the activating NK cell receptors NKG2D and NCRs recognise HIV-1-infected cells are less extensively studied, but involve the recognition of stress-induced or virus-derived cell surface molecules, including MIC-A and MIC-B [82]. As an example, Vpr-induced upregulation of the UL16 binding protein (ULBP)-1, -2 and -3 on infected CD4+ T lymphocytes leads to cytotoxic killing that is NKG2D-dependent [83] (Figure 1C). Taken together, NK cells can use several mechanisms to recognise and kill virus-infected cells, and HIV-1 has evolved mechanisms to counteract NK cell-mediated lysis of infected cells.

### 2.3. NK Cell Cytokine and Chemokine Production

NK cells produce several cytokines and chemokines. Increased cytokine production by NK cells, specifically TNF and IFN-γ, have been associated with protection against HIV-1 infection in HESN IDUs [51]. Similarly, results from serodiscordant couples suggest that higher secretion of IFN-γ by NK cells is involved in the control of HIV-1 establishment in seronegative partners [84]. This cytokine release promotes innate immune cell recruitment and skews dendritic cell (DC) maturation toward a Th1 response, which together could favour the early control of HIV-1 infection [85]. Disease progression, however, positively correlates with higher TNF levels in chronic phase HIV-1 infection [86], plausibly as a consequence of TNF modulation by HIV-1 proteins to enhance viral replication [87,88], suggesting a deleterious effect in the later stages of infection.

NK cells have also been suggested to be involved in the control of HIV-1 replication via the release of β-chemokines CCL3, CCL4 and CCL5. β-chemokines are natural ligands for the HIV-1 co-receptor CC-chemokine receptor 5 (CCR5). Anti-HIV-1 activity is likely exerted by blocking the binding of HIV-1 envelope to CCR5 and thereby preventing viral usage of CCR5 for entry into target cells [89]. In vitro resistance to R5 HIV-1 strains have been demonstrated in PMBC from elite controllers with increased CCL3 levels [90], and natural resistance to HIV-1 infection was associated with higher frequencies of β-chemokine-producing NK cells in HESN individuals [51].

### 2.4. Memory NK Cells and Their Implications in HIV-1 Infection

Although NK cells are conventionally classified as innate, short-lived effector cells, growing evidence challenges the perception of immunological memory as an exclusively adaptive immune trait and offers insight into the development of NK cells with memory-like features that are broadly defined as either antigen-dependent with typical recall or as more generalised cytokine-induced NK cell memory responses [91,92]. NK cell memory-like traits were first observed as contact hypersensitivity responses in hapten-sensitised RAG-1-deficient mouse models that lacked mature T and B lymphocytes [93]. Development and persistence of virus-specific NK cell memory responses were later demonstrated in response to structurally diverse antigens, including HIV-1-derived protein, and was dependent on NK cells expressing CXCR6 [94]. In viral infection models, adaptive NK cells were first described in mouse cytomegalovirus (MCMV) infection, and was largely mediated by NK cells expressing the activating Ly49H receptor that can bind the CMV-encoded glycoprotein m157 [95,96,97]. In human CMV (HCMV) infection, NK cell memory cells have been shown to be characterised by higher expression of NKG2C, CD57 and KIRs for HLA-C interaction, and functionally exhibit superior ADCC [98,99].

HCMV co-infection is highly prevalent in PLWH [100], and thus the reconfiguration of the NK cell repertoire in HIV-1 infection is partially driven by long-lasting CMV-experienced NK cells. In CMV-seropositive individuals, CD56^dim^ NK cells have been shown to transition to a memory-like NK cell phenotype and displayed heightened ADCC to overlapping HIV-1 peptides that persisted in the presence of antiretroviral therapy (ART) [101,102,103,104]. Presence of memory-like NK cell subpopulations have been described to be negatively correlated with HIV-1 viremia and to be associated with lower viral set point during primary HIV-1 infection [105,106], suggestive of a CMV-primed NK cell subset that offers better HIV-1 control. Apart from CMV-primed adaptive NK cells, a recent report described HIV-1-specific NK cell memory as a correlate of HIV-1 control in elite controllers. HIV-1-specific NK cell responses were dependent on NKG2C receptor interaction with HLA-E-presented Gag or Env peptides [107]. Moreover, the inflammatory environment during HIV-1 infection could potentially drive expansion of memory-like NK cells. For example, increased IP-10 levels were correlated with adaptive NK cell expansion in PLWH [104], and HIV-1-induced inflammation was associated with the presence of a CD94+CD56^hi^ NK cell population that displayed heightened cytotoxicity to virus-infected target cells [108] (Figure 1D).

Collectively, the data highlight the diverse NK cell-meditated responses during HIV-1 infection and the counter-mechanisms established by the virus for immune evasion. Clearance of virus-infected cells might be enhanced by augmentation of distinct NK cell functions, including therapeutic expansion of specific NK cell subsets with strong antiviral activity and/or blocking the binding of inhibitory NK cell receptors that serve as checkpoint inhibitors. Furthermore, antibodies directed against HIV-1-infected cells could enhance elimination of infected cells by NK cells through ADCC. Finally, inhibition of viral proteins that enable immune evasion from NK cells may enhance the antiviral potency of NK cells in HIV-1 infection.

## 3. Consequences of HIV-1 Infection on NK Cells

NK cells are key effectors in the immune control of HIV-1 infection, serving as first line antiviral responders and in facilitating the induction of quality adaptive immunity. However, progressive HIV-1 infection profoundly affects the NK cell compartment; most apparent are the permutations in NK cell subset distribution and dysregulations in receptor expression that manifest in defective NK cell responses. Viral suppression with effective ART can alleviate these virus-induced impairments of NK cell function, although to varying degrees that is clearly dependent on the timing of ART initiation, with preferable clinical outcomes subsequent to earlier ART initiation.

### 3.1. NK Cell Count and Subset Redistributions

**Figure 2:**
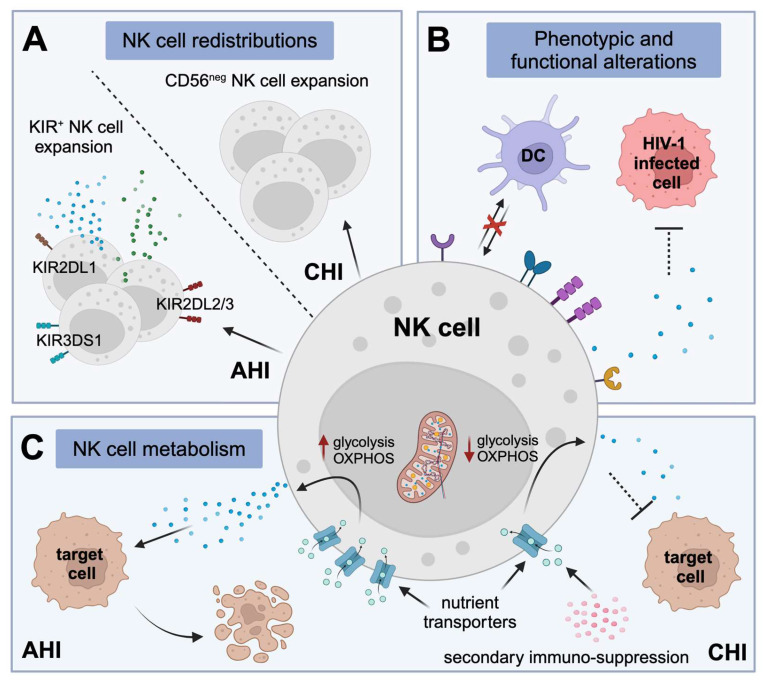
Consequences of HIV-1 infection on NK cells. (**A**) During acute HIV-1 infection (AHI), there is an expansion of KIR+ NK cells with enhanced cytolytic function. Chronic HIV-1 infection (CHI) is associated with the expansion of functionally impaired CD56^neg^ NK cells. (**B**) Phenotypic permutations to NK cells during HIV-1 infection contribute to impaired NK cell cytolytic responses and dysregulated crosstalk between NK cells and dendritic cells (DCs) that negatively affect DC maturation. (**C**) AHI induces nutrient transporter expression, and upregulates glycolysis and mitochondrial OXPHOS that promotes increased NK cell cytolytic activity for effective killing of virus-infected cells. During CHI, secondary immunosuppressive cytokines contribute to the downregulation of nutrient transporters and repression of NK cell metabolic capacities and cytotoxicity.

Acute phase HIV-1 infection is associated with elevated absolute NK cell counts such that the relative proportion of NK cells can account for up to 50% of peripheral blood mononuclear cells, characterised specifically by an expansion of CD56^dim^ NK cells [109]. Further investigation revealed an expansion of KIR+ NK cells [110] that is to some extent dependent on the HLA class I genotype of the respective individual. For example, KIR3DS1+ NK cells can significantly expand in AHI in individuals encoding for HLA-Bw4 genotypes [40], and the functionality of expanding KIR2DL1+ and KIR2DL2/3+ NK cells during primary HIV-1 infection was higher in those individuals encoding for the respective binding HLA-C molecules [111]. Earlier studies report a reduction in CD56-expressing NK cells as HIV-1 infection progressed from acute to chronic disease [112,113], which was initially presumed to be the result of early NK cell death due to ongoing viral replication or preferential trafficking of NK cells to anatomical sites that were abundant in CD4+ T cell targets [114,115]. However, this has since been largely attributed to sequential NK cell subset redistribution, in particular the emergence of the functionally anergic CD56^neg^ subset paralleled by a loss of more cytolytic CD56^dim^ cells [109,116,117,118] (Figure 2A).

The dynamics surrounding NK cell trafficking during HIV-1 infection remain incompletely understood. Previously, NK cell frequencies and distribution were found to be unchanged in the lymph node (LN) in PLWH during the first year of infection. LN NK cells exhibited a phenotypic profile implicative of a superior capacity for activation and infection control. Strikingly, KIR+ NK cells were infrequent within the LN and ineffective KIR+ NK cell recruitment, a consequence of the inferior migratory capacity of KIR+CX3CR1^low^CXCR1^low^ cells, potentially allows for HIV-1 replication in the LN that is largely unrestricted by NK cell-mediated control [119]. A subsequent study described higher expression of homing receptor CCR7 on blood NK cells, although expression was only transiently observed during AHI [110], while more recently, *CX3CR1* and *ICAM2*, genes linked to NK cell trafficking, were reported to be upregulated within the first week of HIV-1 infection [120]. During chronic HIV-1 infection (CHI), CXCR5+ NK cells with higher activation attributes accumulate in the lymphoid tissue. Although CXCR5+ NK cells from HIV-1-infected tissue displayed partially impaired function, frequencies of this cell subset inversely correlated with HIV-1 burden [121]. Importantly, these findings are suggestive of early NK cell infiltration of secondary lymphoid tissues, with potential implications in the establishment and containment of viral reservoir.

### 3.2. NK Cell Phenotypic and Functional Alterations

During early phase HIV-1 infection, expansion of the CD56^dim^ NK cell subset establishes an NK cell pool with higher cytolytic activity [122]. Recently, Kazer and colleagues described the upregulated expression of NK cell cytotoxic genes (*PRF1*, *GZMB*) during Fiebig stage I/II HIV-1 infection in a cohort of young females [120]. The presence of cytotoxic NK cells during this hyperacute stage of infection was associated with long-term viral control [120], corroborating previous findings suggesting that robust NK cell activity within the first 3 months of HIV-1 infection is linked to higher CD4+ T cell count maintenance for two years post-infection in the absence of ART [123].

However, persistent HIV-1 viremia is associated with the redistribution of NK cell subsets, as discussed above, which contributes to defective antiviral responses. Progressive HIV-1 infection induces substantial alterations in the surface receptor profiles and functional activity of NK cells, and these changes are particularly pronounced within the expanding subset of CD56^neg^ NK cells. One marker that has been suggested to be associated with NK cell dysregulation is the decreased surface expression of the inhibitory NKR sialic acid (Sia)-binding immunoglobulin-like lectin 7 (Siglec-7), which appears to be highly sensitive to virus exposure during AHI, prior to CD56 downmodulation. The differential dynamics of Siglec-7 and CD56 receptor expression enables the identification of NK cell subsets that distinguish progressive HIV-1-disease stages; i.e., presence of the Siglec-7^neg^CD56^dim^ NK cell phenotype observed only in AHI followed by the detection of Siglec-7^neg^CD56^neg^ NK cells in CHI [124]. Persistent HIV-1 viremia also diminishes the expression of inhibitory NKG2A receptor [117], which together with the preferential expansion of NKG2C+ NK cells described in CMV infection, skews the NKG2A/NKG2C ratio in PLWH with concomitant HCMV infection [125]. Furthermore, elevated levels of surface inhibitory KIR and reduced activating NCR expression have been reported in HIV-1 viremic compared to aviremic and uninfected individuals [117,126,127]. These phenotypical perturbations observed within NK cell profiles have been associated significantly with an impaired cytotoxic function of NK cells due to relatively high inhibitory receptor expression [118]. Furthermore, the CD56^neg^ NK cell population has been shown to produce higher quantities of transforming growth factor-β (TGF-β), a cytokine associated with homeostatic and anti-inflammatory functions, that may hinder autologous CD8+ T cell responses [128] (Figure 2B).

HIV-1-driven inflammation plays a key role in the expansion of the CD56^neg^ subset. Milush and colleagues previously suggested that this pathological NK cell subset was the consequence of activated mature NK cells that had recently engaged with target cells [129]. The augmented NK cell activation observed in progressive HIV-1 infection is primarily restricted to CD56^dim^ NK cells; and NK cell activation (measured by CD38 and HLA-DR) directly associated with inflammatory mediators, particularly IP-10, and monocyte activation [130]. Importantly, these NK cell aberrancies are not observed in elite controllers [124,130], thus providing important clues to the mechanisms underlying viral control in individuals who naturally suppress HIV-1 viremia.

Under physiological conditions, reciprocal interactions between NK cells and DCs augment DC maturation and their ability to present antigens to T cells. On the other hand, DC-secreted cytokines can activate and expand NK cells, thereby enhancing NK cell-mediated killing of virus-infected cells during acute viral infections [131]. However, HIV-1 infection can perturbate this crosstalk between NK cells and DCs. Lower cytokine production (IL-12, IL-15 and IL-18) by DCs during AHI has direct consequences for IFN-γ production by NK cells. In turn, diminished IFN-γ and TNF production by NK cells negatively affects DC maturation. Moreover, accumulation of CD56^neg^ NK cells hinders the capacity of NK cells to execute DC-editing [109,118,132]. Increased IL-10 production, characteristic in CHI, prompts the induction of tolerogenic DCs through direct and indirect mechanisms [133,134], including this dysregulation of NK cells in their crosstalk with DCs. Lastly, the HIV-1 proteins Tat and Nef have been implicated in impairing the crosstalk between NK cells and DCs [135] (Figure 2B). Collectively, these data demonstrate NK cell dysregulation in HIV-1 infection, with consequences for effective innate immune crosstalk that can result in an impairment of adaptive immunity, which ultimately lead to defective viral control and disease progression.

### 3.3. The Effect of ART on HIV-Induced NK Cell Dysregulation

Effective suppression of HIV-1 viremia leads to partial immune restoration that varies with the timing of ART administration [136,137,138,139,140,141]. Previous reports suggest moderate improvement of NK cell subset distribution, surface molecule expression and cytotoxic responses after ART initiation. Gradual recovery of Siglec-7 and CD56 expression on NK cells was observed within 24 months on suppressive therapy [124], and ART alone or in combination with IL-2 therapy promoted CD56^dim^ NK cell expansion [142,143]. Similarly, normalisation of the NKG2A/NKG2C ratio required at least 24 months of undetectable HIV-1 replication [125]. Expression of inhibitory NKRs and NCRs are for the most part reversed to levels comparable to that of uninfected individuals [117], whilst other markers of NK cell activation, including CD69 and CD38/HLA-DR co-expression, seemingly persist in ART-positive patients, independent of HIV-1 viremia, CD4+ T cell count or T cell activation [144,145]. However, one study, which attributes increased NK cell activation to CD56^dim^ NK cells in immunological non-responder PLWH, found NK cell activation to inversely associate with recovery of CD4+ T cells [146]. ART-induced restoration of NK cell activating and inhibitory receptor profiles correlated with NK cell cytotoxicity [117], and enhanced terminal differentiation of CD56^dim^ NK cells was suggestive of functionally mature NK cells post-ART initiation [147]. Furthermore, rescue of NK cell-mediated ADCC was more pronounced when ART was initiated prior to seroconversion [148]. Importantly, the positive effects of ART on other immune subsets augments recovery of NK cell responses; specifically, the proper maturation and restoration of cytokine production by DCs in PLWH favourably influences NK cell priming and IFN-γ production [149]. Taken together, suppressive ART can partially reverse HIV-induced NK cell defects; however, persistent NK cell activation possibly contributes to chronic inflammation and risk for comorbid diseases.

### 3.4. Cellular Metabolism and NK Cell Functionality in HIV-1

Cellular metabolism is a critical factor in the fate of immune cells; the key outputs of which contribute to homeostasis, energy production and the regulation of biosynthetic precursors that are essential for cellular processes. These metabolic processes are sustained primarily by two interlinked pathways: glycolysis and mitochondrial oxidative phosphorylation (OXPHOS). Accumulating evidence describes the indispensable link between cellular metabolism and the modulation of immune effector functions, with implications for immune responses during times of metabolic dysregulation [150]. NK cell homeostasis is sufficiently supported by relatively low rates of OXPHOS, which during short-term activation can sustain NK cell function without significant metabolic modulation [151,152,153]. Longer periods of exposure to stimuli induce “metabolic reprogramming” in NK cells to incorporate the upregulation of both glycolysis and OXPHOS to support the growing energy demands needed for the maintenance of NK cell function. Although mitochondrial OXPHOS is a more efficient pathway for ATP generation, a shift towards glycolysis is integral for rapid production of ATP, cellular proliferation and the generation of precursors that are necessary for the synthesis of immune effector molecules [154,155].

The metabolic dynamics adopted by individual immune cells are further determined by the energy requirements relating to cell type, functional phenotype and the differential impact of extrinsic cues. Increased cellular metabolism is supported by enhanced nutrient transporter expression to facilitate an influx of nutrient sources and an upregulation of metabolic enzymes [9,151,152,156,157]. In particular, glucose is a major fuel for cellular metabolism in lymphocytes, as evidenced by augmented glucose transporter 1 (GLUT1) expression and uptake of glucose upon activation [151,156,158]. Apart from glucose metabolism, other fuel sources include glutamine and fatty acids, both of which are converted to intermediates that downstream supply mitochondrial respiration. Cytokine stimuli induces higher expression of the L-type amino acid transporter CD98 on NK cells [9,157], whilst data pertaining to the contribution of fatty acid oxidation to NK cell metabolism are currently lacking. The availability of nutrients within the local microenvironment, such as tissues, can be sensed by intracellular metabolic regulators that subsequently affect the modulation of metabolic pathways. As such, nutrient excess or deprivation greatly influences metabolic plasticity with consequences for cellular fate [159,160].

Recent studies investigating immune metabolism of NK cell subsets have started to reveal the distinct metabolic pathways that are preferential to specific immune outputs. In peripheral blood, the CD56^bright^ NK cell subset exhibited superior metabolic activity in comparison to CD56^dim^ cells [153]. In response to cytokine stimulus, CD56^bright^ NK cells upregulated nutrient transporters and glucose metabolism, but were more susceptible to metabolic inhibition [153]. Nutrient receptor profiles of NK cell subsets are further influenced by compartment localisation. For example, peripheral blood CD56^bright^ NK cells displayed higher GLUT1 expression indicative of glucose metabolism relative to matched tissue-resident CD56^bright^ cells that preferentially expressed L-type amino acid transporter CD98, indicating the capacity for metabolic adaptation in response to nutrient variability within the respective microenvironment [9]. Moreover, functional dependency on cellular metabolism is emphasised by the distinct metabolic reprogramming observed between uneducated and educated NK cells. Murine models demonstrated the significance of metabolic regulator mammalian target of rapamycin (mTOR) activity in the attainment of cytotoxic potential during NK cell development [151]. These findings were later confirmed in NK cells sorted for uneducated and educated NK cell populations from KIR/HLA-genotyped individuals [161]. Functional activities subsequent to cellular activation relied solely on OXPHOS in uneducated NK cells, whilst licensed NK cells engaged in both glycolysis and mitochondrial-dependent glutaminolysis for optimal cytotoxic function [161].

Assessment of glycolytic and mitochondrial respiratory pathway suppression with the inhibitory drugs, 2-deoxyglucose and oligomycin, respectively, has begun to deduce the intricacies of metabolic pathway regulation during cellular activation that directly impacts immune cell function. Inhibition of either glycolysis or OXPHOS led to lower IFN-γ production by NK cells, whereas selective suppression of glycolysis reduced degranulation marker and Fas ligand expression associated with NK cell-mediated cytotoxicity in the context of NKR/ligand-induced activation [152,162]. Furthermore, the activity of mTOR, which is central to the maintenance of glycolytic pathways, has been investigated. During cellular activation, enhanced mTOR activity results in increased glucose uptake and glycolysis upregulation, promoting the production of IFN-γ and granzyme B effector molecules in NK cells. Rapamycin-induced inhibition of mTOR activities directly limited glycolysis, resulting in inferior NK cell–antiviral responses [153,156].

Chronic viral infections induce NK cell impairments that have been well characterised; however, data pertaining to the metabolic changes linked to these functional impairments are not well defined. CMV remains the most common model for understanding NK cell biology in the context of viral infection. MCMV infection studies demonstrated induction of nutrient transporter expression on NK cells to support nutrient uptake, gene upregulation associated with aerobic glycolysis, and higher oxygen consumption and extracellular acidification rates indicative of increased cellular respiration [163,164]. Inhibition of glucose metabolism in MCMV-infected mice reduced NK cell-mediated clearance of target cells and significantly increased susceptibility to MCMV-induced mortality [165]. Similarly, NK cell-specific mTOR suppression negatively impacted cytokine responses during early MCMV infection [151] and deletion of the lactate dehydrogenase A (LDHA) enzyme, which is central to glycolysis signalling, resulted in defective NK cell antiviral protection [163]. In humans, HCMV-induced adaptive NK cells exhibited more pronounced mitochondrial OXPHOS and spare respiratory capacity compared to canonical NK cells [166], plausibly to support specialised effector and memory functions.

Although studied less extensively, the modification of NK cell metabolism induced by HIV-1 infection likely bear similarities to those observed for other chronic viral infections where metabolism defects lead to functional impairment of NK cells [167]. HIV-1-induced secondary immunosuppression, which is biologically intended to counteract persistent systemic inflammation in HIV-1 infection, is largely implicated in compromising effector immunity and predisposing PLWH to non-AIDS-related morbidities. In particular, the anti-inflammatory cytokine TGF-β has been identified as an important contributor to HIV-1-associated immunosuppression, and TGF-β is persistently elevated in PLWH, even when on suppressive ART [168,169]. In vitro studies have implicated TGF-β activity in the inhibition of cytokine-induced NK cell nutrient transport expression, repression of NK cell metabolic capacities and reduced NK cell cytotoxicity [170,171]. Importantly, suppression of the TGF-β signalling pathway was shown to lead to both NK cell metabolic restoration and improved functional responses [171]. Recently, adaptive NK cells from HCMV-experienced PLWH showed a loss of the bioenergetic advantages that is characteristic of these adaptive NK cells during CHI. Instead, phenotypic CD57+NKG2C+ NK cells exhibited structural and functional mitochondrial defects, which notably could be alleviated with IL-15 pre-treatment [172] (Figure 2C). Taken together, these data are suggestive of the virus-induced metabolic defects of canonical and adaptive NK cells during chronic viral infection, including HIV-1 infection, that associate with NK cell dysregulation, and that could be compensated for through pharmacological metabolic reprogramming.

Overall, the relationship between cellular metabolism and immune function has prompted accelerated research in the field of immune cell metabolism, and there is clear evidence of metabolic perturbations that are associated with reduced NK cell functions. However, in-depth investigations are warranted to elucidate the intricate mechanisms underpinning NK cell metabolism and their relation to NK cell differentiation and function. Nonetheless, modulation of NK cell metabolism could represent new targets for improved NK cell functions during viral infections.

## 4. Conclusions

Suppression of viral replication by ART has significantly reduced HIV-1-associated morbidity and mortality in PLWH. However, life-long ART is required and is associated with elevated immune activation and residual immune dysfunctions, emphasising the need for efficacious immunotherapies to reconstitute immune function. NK cells are potent anti-HIV-1 immune cells, and the emerging understanding of their functional diversity now offer several potential approaches to leverage NK cells that, in conjunction with adaptive immunity, could be harnessed for therapeutic intervention in HIV-1. These interventions would essentially encompass a multifaceted approach that enhances both NK cell antiviral and immunoregulatory functions. In particular, enhanced ADCC responses that are directed at key viral reservoir sites is a promising strategy for improving viral control. However, CHIinduces permutations of NK cell populations and their functions that are only partially restored by ART. Increasing data have elucidated the critical link between cellular metabolism and the immune function of NK cells and identifying novel metabolic pathways that alleviate NK cell impairment might serve as targets for therapies that reconstitute and enhance NK cell functions in PLWH.
